# Optimal barbell force-velocity profiles can contribute to maximize weightlifting performance

**DOI:** 10.1371/journal.pone.0290275

**Published:** 2023-08-18

**Authors:** Ingo Sandau, Urs Granacher

**Affiliations:** 1 Department of Strength, Power and Technical Sports, Institute for Applied Training Science, Leipzig, Germany; 2 Department of Sport and Sport Science, Exercise and Human Movement Science, University of Freiburg, Freiburg im Breisgau, Germany; University of Vic - Central University of Catalonia: Universitat de Vic - Universitat Central de Catalunya, SPAIN

## Abstract

Maximal barbell power output (*P*_max_) and vertical barbell threshold velocity (*v*_thres_) are major determinants of weightlifting performance. Moreover, an optimal force-velocity relationship (FvR) profile is an additional variable that has the potential to maximize sports performance. The aims of this study were (i) to present a biomechanical model to calculate an optimal FvR profile for weightlifting, and (ii) to determine how *v*_thres_, *P*_max_, and the optimal FvR profile influence theoretical snatch performance (*snatch*_th_). To address these aims, simulations were applied to quantify the respective influence on *snatch*_th_. The main findings confirmed that at constant *v*_thres_ and *P*_max_, *snatch*_th_ is maximized at an optimal FvR profile. With increasing *P*_max_ and decreasing *v*_thres_, the optimal FvR profile becomes more force dominated and more effective to enhance *snatch*_th_. However, sensitivity analysis showed that *v*_thres_ and *P*_max_ have a larger effect on *snatch*_th_ than the optimal FvR profile. It can be concluded that in weightlifting, training protocols should be designed with the goal to improve *P*_max_ and to reduce *v*_thres_ to ultimately enhance *snatch*_th_. Training programs designed to achieve the optimal FvR profile may constitute an additional training goal to further develop weightlifting performance in elite athletes that already present high *P*_max_ levels.

## Introduction

In ballistic sports such as track and field or weightlifting, performance is ultimately determined by the athlete´s capability to maximally accelerate their body mass (e.g., sprinting, jumping) or a maximal external load (i.e., weightlifting) [1, 2]. In all of these examples, the velocity reached at the end of the propulsive phase is related to mechanical output parameters produced through efficient work of the neuromuscular system [3]. In this context, a linear force-velocity relationship (FvR) has frequently been used to assess mechanical parameters such as the theoretical maximal velocity at zero force (*v*_0_) and the theoretical maximal force at zero velocity (*F*_0_). From *v*_0_ and *F*_0_, maximal power output (*P*_max_) and the FvR profile (i.e., slope of the FvR; *s*_FvR_) can be computed [4]. Previous research has shown that *P*_max_ is a major determinant of maximal ballistic performance [5, 6]. In addition, Samozino and colleagues further reported that for a given *P*_max_ level, performance in vertical jumping and sprinting is (theoretically) maximized at an optimal FvR profile [4, 7, 8]. Accordingly, a customized resistance training program to improve ballistic sports performance should aim to maximize *P*_max_ through optimization of the FvR profile. Although the benefits of an optimal FvR profile for practical use are still under debate [9], there is partial evidence that resistance training programs designed to optimize the FvR profile were more successful than standard (i.e., non-optimized) resistance training protocols in improving vertical jump height in trained soccer, rugby, and futsal player [10–12].

In weightlifting, maximal performance is determined by the lifters neuromuscular capabilities to produce a high power output combined with well-developed lifting technique (e.g., turnover and catch phase) [13]. The technical mastery and effectiveness of the lift is reflected by the individual’s vertical threshold velocity (*v*_thres_) [14]. The v_thres_ is the minimum peak vertical barbell velocity (*v*_max_) an individual athlete needs to lift a maximal barbell load successfully in the overhead position. For example, the *v*_max_ during a one-repetition maximum (1RM) lift in the snatch is denoted as *v*_thres_ in the snatch. In general, *v*_thres_ ensures a necessary vertical travel distance and flight time of the maximal barbell load (i.e., projectile motion) at the end of the acceleration phase, which allows the athlete to squat under and catch the barbell in the overhead position.

Consequently, weightlifters who have better technical skills in the turnover and catch phase can lift with lower levels of *v*_thres_. For example, better technical skill performance is indicated through larger amounts of “residual work” and a shorter time-span of the turnover phase [14, 15]. term “residual work” has previously been defined as the vertical distance the barbell travels beyond the theoretical distance from projectile motion (i.e., v_max_) due to an acting vertical force component that is initiated through the upper extremities [14]. In addition, a low *v*_thres_ corresponds to a smaller amount of force that needs to be utilized to accelerate the barbell. Hence, higher force levels can be achieved to overcome gravitational forces which results in better weightlifting performance. As recently presented, weightlifting is a good example to use FvR-parameters (i.e., *F*_0_, *v*_0_, *P*_max_) to monitor progression during training and to predict weightlifting performance [16, 17]. In agreement with results from vertical jumping [4], for individual time-series data *P*_max_ has been shown to be highly–but not perfectly (i.e., cross-correlation coefficients range from 0.86–0.88)–associated with the theoretical snatch performance (*snatch*_th_) in elite weightlifters [17]. Due to the imperfect correlation of *snatch*_th_ and *P*_max_, the specific FvR profile was assumed to be another determinant of weightlifting performance [17] as it has been previously presented for the vertical jump [4]. Accordingly, a given *P*_max_ level in combination with an optimal FvR profile may maximize snatch performance.

Therefore, the main aim of this study was to determine how the optimal FvR profile (if any), *v*_thres_, and *P*_max_ influence weightlifting performance. Considering the aforementioned relation between maximal vertical jump performance and an optimal FvR profile [7], we hypothesized that for a given level of *P*_max_ and *v*_thres,_ the FvR profile has an impact on the theoretical snatch performance (H1), and that the theoretical snatch performance is maximized at an optimal FvR profile (H2). Further, we were interested to elucidate the extent to which *P*_max_, the FvR profile, and *v*_thres_ influence *snatch*_th_. With reference to the literature [4, 14], we hypothesized that changes in *P*_max_ and *v*_thres_ influence *snatch*_th_ to a larger degree compared with changes of the FvR profile (H3).

To address these aims, first, we present the biomechanical concept of optimal FvR profile applied to weightlifting (i.e., snatch pull model) from which the maximal theoretical snatch performance (${snatch}_{th}^{max}$) can be computed. In the second part, we used mathematical simulations to apply the snatch pull model with data from the literature. The simulations were used to quantify the influence of the optimal FvR profile, *v*_thres_, and *P*_max_ on *snatch*_th_.

## Methods

### Theoretical background

This section is dedicated to the theory of optimal FvR profile and how an optimal FvR profile may positively influence weightlifting performance. For this purpose, we briefly recap the established biomechanical model from vertical jump and transfer it to weightlifting.

In a linear modelled FvR profile from loaded vertical jumps, *P*_max_ is located at 0.5*F*_0_ and 0.5*v*_0_, respectively [3]. Consequently, *P*_max_ can be calculated as:

$$P_{max}={0.25\;v}_{0}{\bar{F}}_{0}.$$ (1)

In this context, the vertical force at *P*_max_ (i.e., 0.5*F*_0_) is associated with an external load condition during the jump. For a given FvR profile, the load at *P*_max_ conditions is interpreted as the optimal load [18]. In case of a vertical jump, when *P*_max_ is located at a load that corresponds to the jumpers body mass (i.e., body mass = optimal load) an optimal FvR profile is achieved [4]. Under optimal FvR profile conditions (i.e., *P*_max_ is located at body mass), the vertical take-off velocity of the body´s center of mass–and hence the maximal jump height–is maximal. In contrast, even at constant *P*_max_ level, with a non-optimal FvR profile (i.e., *P*_max_ is located at loads smaller or larger then body mass), the achieved vertical take-off velocity is less than the maximal vertical take-off velocity at an optimal FvR profile. In other words, with a non-optimal FvR profile, the actual external mechanical power-output during a jump is less than the jumper’s maximal power capacities (*P*_max_) [19]. The concept of delivering *P*_max_ at the load condition corresponding to the targeted movement (here unloaded vertical jump) can be used as a starting point to find an optimal FvR profile that maximize performance in weightlifting.

### Transferring theory from jumping to establishing optimal barbell FvR profiles in weightlifting

**Fig 1:**
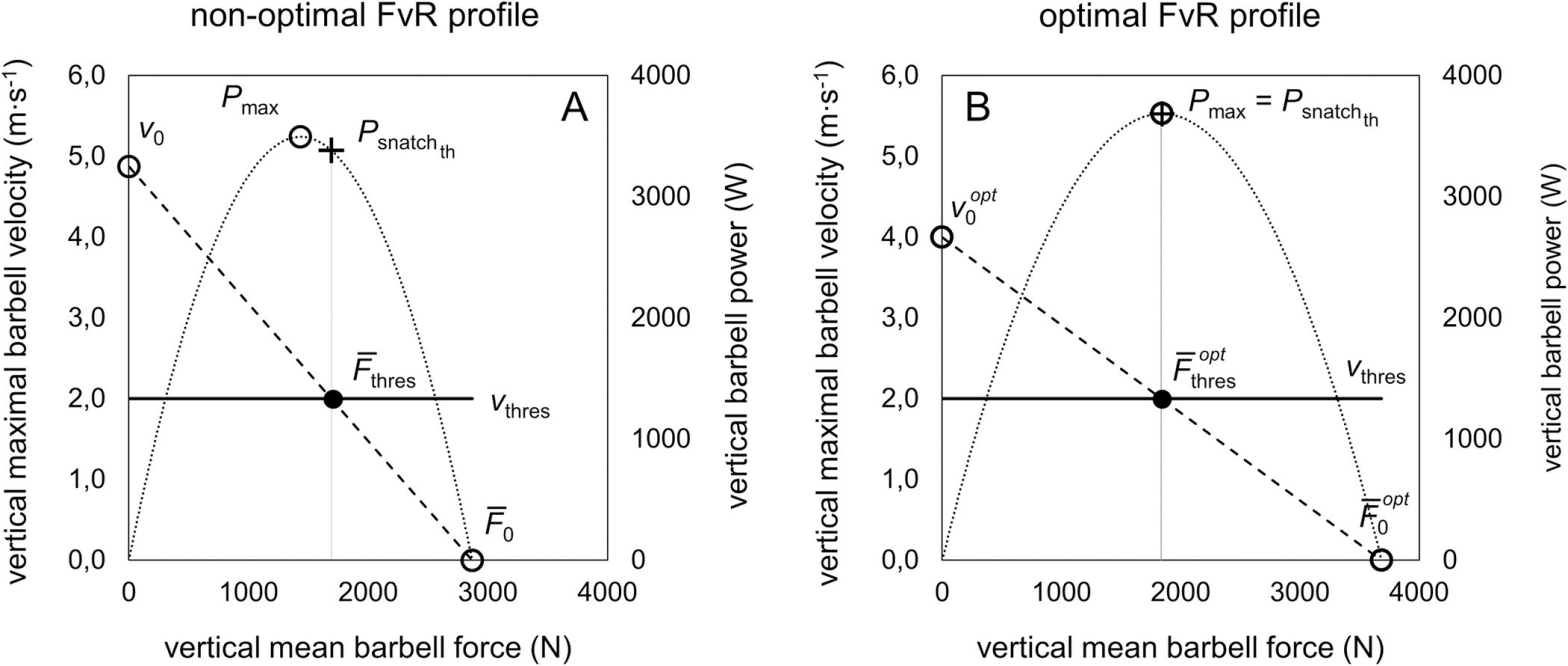
Graphical example of two barbell force–velocity relationships (FvR) (dashed black lines) with corresponding barbell power outputs (dotted black lines) representing non–optimal (A) and optimal profiles (B) during the specific snatch pull test (*P*_max_ = maximal vertical barbell power, *v*_0_ = theoretical maximal vertical barbell velocity at zero mean barbell force, ${\bar{F}}_{0}$ = theoretical maximal vertical mean barbell force at zero maximal vertical barbell velocity, $P_{{snatch}_{th}}$ = vertical barbell power at *snatch*_th_, ${\bar{F}}_{\mathrm{thres}}$ = vertical mean barbell force at *snatch*_th_, ${\bar{F}}_{thres}^{opt}$ = optimal mean barbell force at ${snatch}_{th}^{max}$, *v*_thres_ = individual vertical barbell threshold velocity for 1RM snatch, opt = optimal).

Performance in weightlifting is simply defined as the maximal load that can be lifted in the snatch and the clean and jerk [20]. However, a successful maximal lift requires acceleration of the barbell load up to an individual *v*_thres_ during the acceleration phase [2, 14]. As previously outlined for the vertical jump [21], performance in weightlifting can also be described by two interacting constraints: i) movement specific barbell velocity conditions (i.e., *v*_thres_ in m∙s^-1^) (black line in Fig 1A) and ii) mechanical output produced by the neuromuscular system (i.e., barbell FvR; dashed black line in Fig 1A).

In this context, it has recently been shown, that for the individual weightlifter, the theoretical snatch performance (*snatch*_th_ in kg) can be calculated from the linear snatch pull FvR profile and the known *v*_thres_ of a 1RM snatch [16]. For example, *v*_thres_ is a highly individual constant that can be obtained from 1RM snatches in competitions using video-based analysis of barbell kinematics. The interaction of the two aforementioned mechanical constraints can be visualized by the intersection of the two lines, giving the mean barbell force at *v*_thres_ (${\bar{F}}_{\mathrm{thres}}$ in N) (black dot in Fig 1A) from which *snatch*_th_ can be calculated. According to the approach outlined by Sandau et al. [16], to compute *snatch*_th_, first ${\bar{F}}_{\mathrm{thres}}$ needs to be computed from *v*_thres_, *v*_0_ (in m∙s^-1^), and *s*_FvR_ (in m∙s^-1^∙N^-1^) as follows:

$${\bar{F}}_{thres}=\frac{v_{thres}-v_{0}}{s_{FvR}}.$$ (2)

Of note, as adjustment to the approach proposed by Sandau et al. [16], in Eq (2) *v*_0_ and *s*_FvR_ were extracted from a snatch pull FvR modelled with maximal (instead of mean) vertical barbell velocity (ordinate) and mean vertical barbell force (abscissa). Therefore, the slope of the FvR is calculated as:

$$s_{FvR}=-\frac{v_{0}}{{\bar{F}}_{0}}.$$ (3)

${\bar{F}}_{\mathrm{thres}}$ represents the sum of the barbell force due to gravity (*g* in m∙s^-2^) and the barbell force due to the mean vertical barbell acceleration to achieve *v*_thres_ (${\bar{a}}_{\mathrm{thres}}$ in m∙s^-2^). Consequently, *snatch*_th_ is obtained by:

$${snatch}_{th}=\frac{{\bar{F}}_{thres}}{g+{\bar{a}}_{thres}}.$$ (4)

In Eq (3), ${\bar{a}}_{\mathrm{thres}}$ can be calculated using *v*_thres_ and the vertical distance of barbell acceleration (i.e., *h*_acc_ in m; vertical height of the barbell at the instance of *v*_thres_ minus the radius of barbell plates [0.225 m]):

$${\bar{a}}_{thres}=\frac{{v_{thres}}^{2}}{2h_{acc}}.$$ (5)

Substitution Eqs (1) and (4) in Eq (3), and after simplification, *snatch*_th_ can be expressed as:

$${snatch}_{th}=\frac{2h_{acc}(v_{thres}-v_{0})}{s_{FvR}(2gh_{acc}+v_{thres}{}^{2})}.$$ (6)

To express *snatch*_th_ as a function of *s*_FvR_ and *P*_max_, using Eqs (1) and (3), *v*_0_ can be calculated as:

$$v_{0}=2\sqrt{-P_{max}s_{FvR}}.$$ (7)

Finally, substituting Eq (7) in Eq (6) gives:

$${snatch}_{th}=\frac{2h_{acc}(v_{thres}-2\sqrt{-P_{max}s_{FvR}})}{s_{FvR}(2gh_{acc}+v_{thres}{}^{2})}$$ (8)

As mentioned above, maximal ballistic performance is achieve, when the actual external power-output during a movement equals *P*_max_. For the individual weightlifter, the actual vertical power output during *snatch*_th_ is highly associated with the absolute load of *snatch*_th_ [17]. Since power is the product of force and velocity, using Eq (2), the actual vertical power output during *snatch*_th_ ($P_{{snatch}_{th}}$ in W) can be calculated as:

$$P_{{snatch}_{th}}={\bar{F}}_{thres}v_{thres}$$ (9)

With ${\bar{F}}_{\mathrm{thres}}$ being the mean vertical barbell force at *snatch*_th_ (Eq (2)). Substituting Eq (2) in Eq (9) gives:

$$P_{{snatch}_{th}}=\frac{v_{thres}{-v}_{0}}{s_{FvR}}v_{thres}$$ (10)

As can be seen for the exemplary barbell FvR profile in Fig 1A, $P_{{snatch}_{th}}$ is not located at *P*_max_. Following the same mechanical principle presented for the vertical jump, a mismatch of $P_{{snatch}_{th}}$ and *P*_max_ can be interpreted as a non-optimal barbell FvR profile that results in a non-maximal *snatch*_th_. In this case, only a fraction of *P*_max_ can be used to accelerate the barbell load to *v*_thres_. In turn, an optimal barbell FvR profile that enables the weightlifter to accelerate the barbell with the maximal possible vertical barbell power output (*P*_max_) may result in a maximized *snatch*_th_ performance (${snatch}_{th}^{max}$). Again, even with a given *P*_max_ level, *snatch*_th_ is maximized at an optimal *s*_FvR_ ($s_{FvR}^{opt}$). Graphically, this $s_{FvR}^{opt}$ is achieved, if *P*_max_ is exactly located at $P_{{snatch}_{th}}$ (Fig 1B). In the presented example, $s_{FvR}^{opt}$ is reached when *P*_max_ is shifted towards $P_{{snatch}_{th}}$ (to the right) by an increase in ${\bar{F}}_{0}$ and a decrease in *v*_0_. In other words, the non-optimal *s*_FvR_ in this case is caused by a force “deficit” or a velocity “surplus”, respectively. For example, if *P*_max_ needs to be shifted to the left to match $P_{{snatch}_{th}}$, the non-optimal *s*_FvR_ is caused by a velocity “deficit” or a force “surplus”, respectively.

As previously mentioned for the optimal FvR profile in vertical jumps, under $s_{FvR}^{opt}$ conditions, *v*_thres_ equals $0.5v_{o}^{opt}$ and ${\bar{F}}_{thres}^{opt}$ equals $0.5{\bar{F}}_{0}^{opt}$. Since *v*_thres_ is an individual known constant for a 1RM snatch lift, $v_{o}^{opt}$ can be simply calculated as:

$$v_{0}^{opt}=2v_{thres}$$ (11)

Using Eqs (2) and (3), and after simplification, the $s_{FvR}^{opt}$ can be calculated as:

$$s_{FvR}^{opt}=-\frac{v_{thres}^{2}}{P_{max}}$$ (12)

Finally, ${snatch}_{th}^{max}$ is obtained by substituting Eq (12) in Eq (8).

### Model simulation

In the theoretical background, we presented a biomechanical model from which the optimal FvR profile (i.e., $s_{FvR}^{opt}$) and the maximal theoretical *snatch*_th_ (${snatch}_{th}^{max}$) can be computed. This model is based on a snatch pull linear two-point FvR (i.e., only two loading conditions were applied during the snatch pull test) that is obtained using linear regression in eight elite male weightlifters [22]. In order to illustrate how changes in *P*_max_ and *v*_thres_ may moderate $s_{FvR}^{opt}$ and ${snatch}_{th}^{max}$, typical ranges of *P*_max_ and *v*_thres_ were applied for simulations using Eq (8). Furthermore, the simulations were used to quantify the respective contribution of *P*_max_, *s*_FvR_, and *v*_thres_ on *snatch*_th_.

For the applied simulations, no new experimental data were collected. Instead, we used data from previous (published) studies of our research group [16, 17, 22]. In total, these experiments were conducted with 10 elite male and 3 elite female weightlifters, modeling the barbell FvR profile and *snatch*_th_ using the aforementioned snatch pull test. From these experiments, typical value for *P*_max_ range from 2000 to 4000 W, for *s*_FvR_ from -0.0005 to -0.0025 m∙s^-1^∙N^-1^, and *h*_acc_ was on average 0.8 m [16, 17, 22]. In addition, the magnitudes of maximal vertical barbell velocities during the 1RM snatch (i.e., *v*_thres_) can also be obtained from the literature. Typical values for *v*_thres_ during the snatch 1RM range from 1.70 to 2.0 m∙s^-1^ [15, 23, 24].

First, changes in ${snatch}_{th}^{max}$ and $s_{FvR}^{opt}$ were analyzed for different *P*_max_ (at constant value of *v*_thres_) and *v*_thres_ values (constant value of *P*_max_), and as a variation of both variables. Second, the influence of *v*_thres_, *P*_max_, and *s*_FvR_ on *snatch*_th_ were analyzed through sensitivity analysis. Within the sensitivity analysis, the relative (i.e., percentage) variation of each independent variable was plotted against the relative *snatch*_th_ change to assess the relative importance of each variable. Although *h*_acc_ has an influence on *snatch*_th_, this variable was treated as a constant in the simulations as it depends on the athlete’s anthropometric characteristics that cannot be influenced through training.

## Results

### Influence of *P*_max_ and *v*_thres_ on *snatch*_th_ and *s*_FvR_

**Fig 2:**
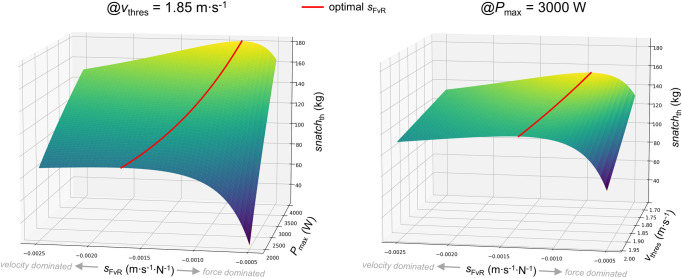
Changes in theoretical 1RM snatch performance (*snatch*_th_) as a function of the slope of the barbell force–velocity relationship (*s*_FvR_) for different values of vertical maximal barbell power (*P*_max_) at a fixed vertical barbell threshold velocity (*v*_thres_) of 1.85 m∙s^–1^ (left), and for different values of *v*_thres_ at a fixed *P*_max_ of 3000 W. The distance of vertical barbell acceleration (*h*_acc_) was set to 0.8 m. The red line represents ${snatch}_{th}^{max}$ at $s_{FvR}^{opt}$.

The simulated influence of *P*_max_ and *v*_thres_ on *snatch*_th_ and *s*_FvR_ is depicted in Fig 2. Findings from the simulation study showed that both *P*_max_ and *v*_thres_ influence *snatch*_th_. Furthermore, at high *P*_max_ or a low *v*_thres_ levels, changes in *s*_FvR_ have a larger potential to moderate *snatch*_th_ due to the more prominent apex of the *snatch*_th_-*s*_FvR_-function (Fig 2). As mathematically presented, *snatch*_th_ is maximized (i.e., ${snatch}_{th}^{max}$) at an optimal value of *s*_FvR_ (i.e., $s_{FvR}^{opt}$, red lines in Fig 2).

**Fig 3:**
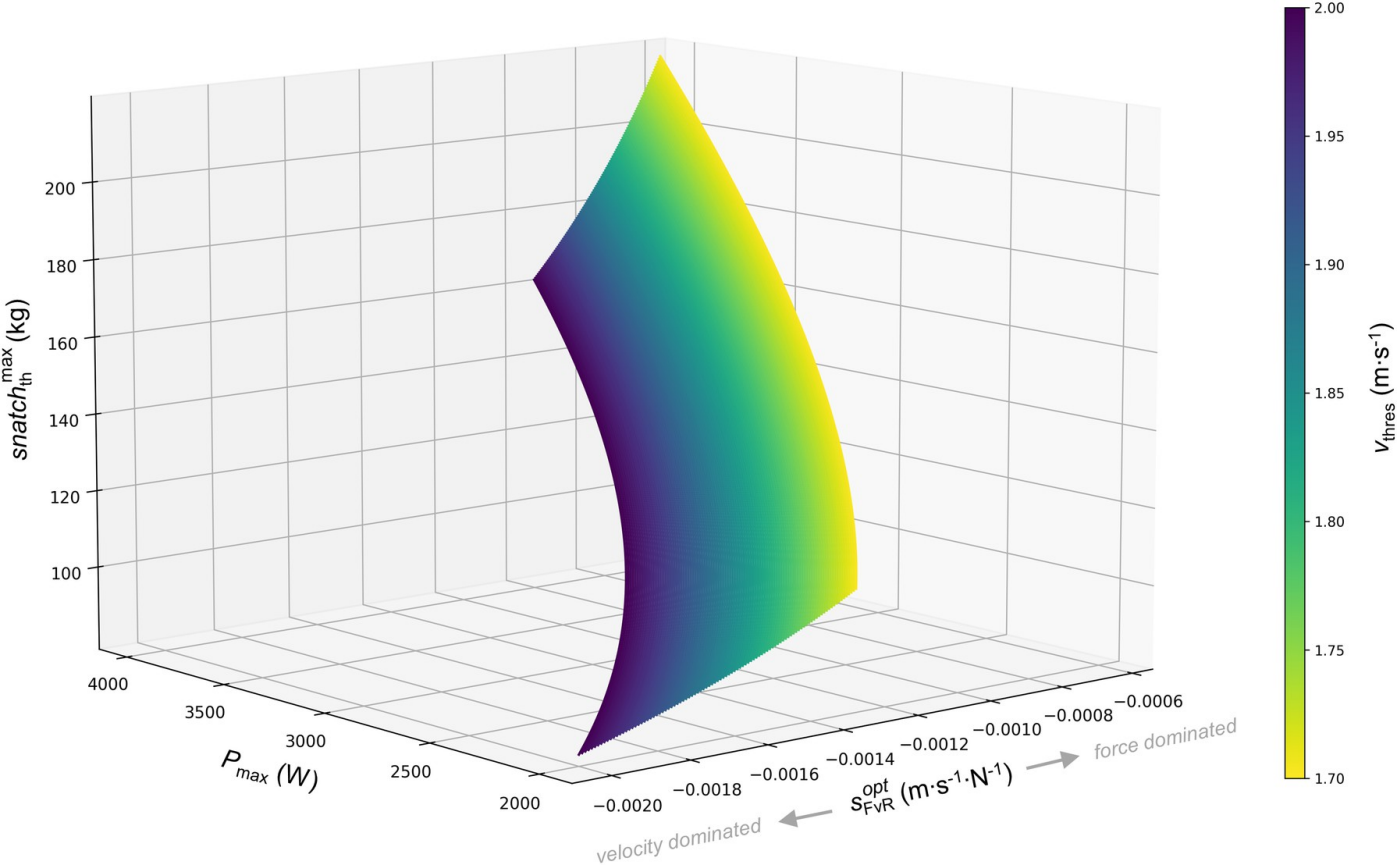
Changes of maximal theoretical 1RM snatch performance (${snatch}_{th}^{max}$) and the optimal slope of the barbell force−velocity relationship $\left(s_{FvR}^{opt}\right)$ as a function of the threshold velocity (vthres) and vertical maximal barbell power (*P*_max_). The distance of the vertical barbell acceleration (*h*_acc_) was set to 0.8 m.

In fact, it is obvious that with increasing *P*_max_ and decreasing *v*_thres_, $s_{FvR}^{opt}$ is shifted towards a more force dominated FvR profile (Figs 2 and 3).

**Fig 4:**
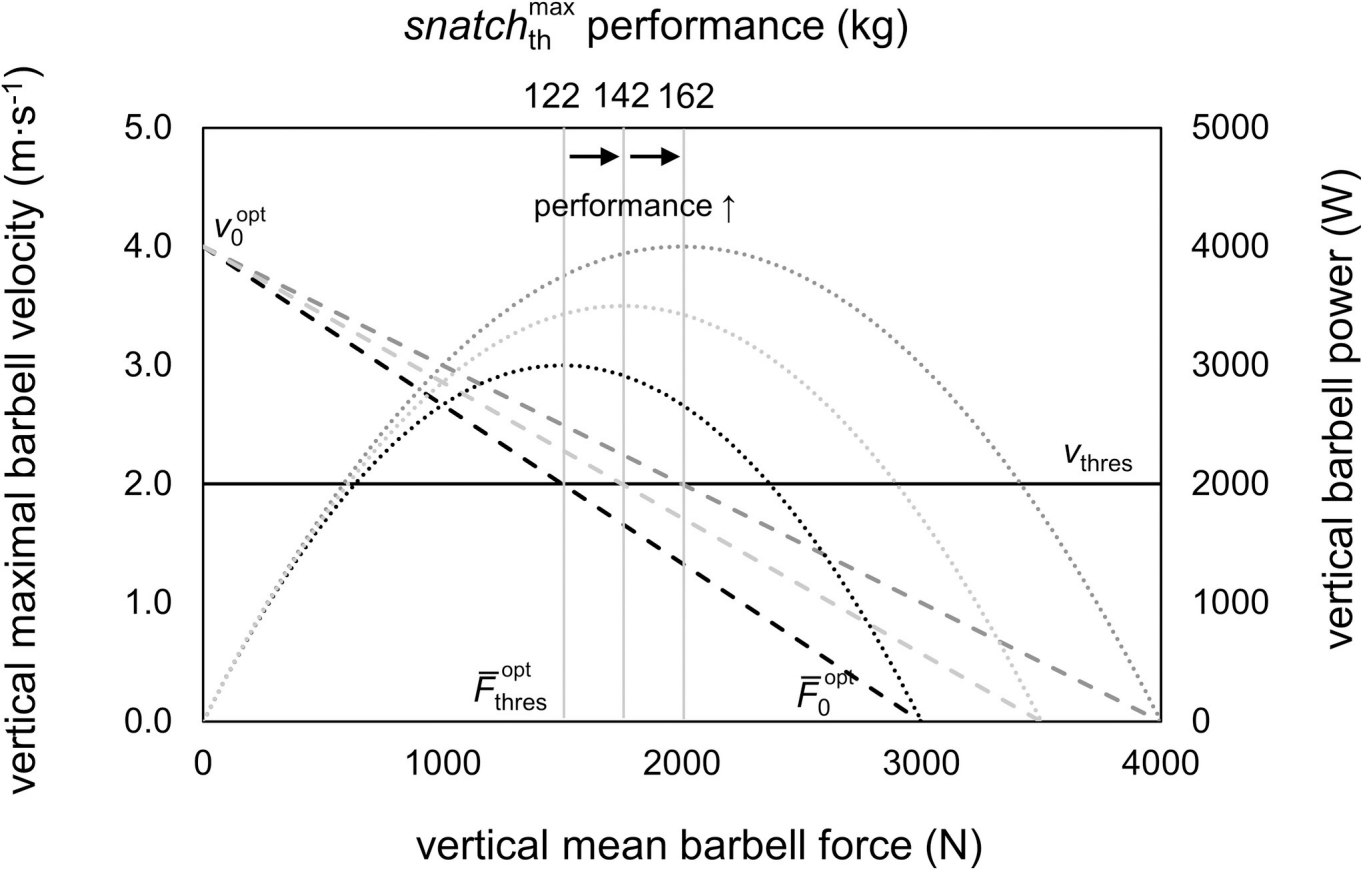
Theoretical model for athlete specific improvements in snatch performance at a fixed theoretical optimal maximal vertical velocity ($v_{0}^{opt}$) of 4.0 m∙s^–1^, a fixed vertical acceleration distance (*h*_acc_) of 0.8 m, and constant vertical threshold velocity of 1RM snatch (*v*_thres_) of 2.0 m∙s^–1^ at optimal barbell FvR conditions. Increased theoretical maximized snatch performance (${snatch}_{th}^{max}$) as a result of increased theoretical optimal maximal vertical mean force (${\bar{F}}_{0}^{opt}$) from 3000 N (black dashed line) to 3500 N (light grey dashed line) to 4000 N (grey dashed line) and the associated increase in maximal power (*P*_max_) from 3000 W (black dotted line) to 3500 W (light grey dotted line) to 4000 W (grey dotted line).

Since $v_{0}^{opt}$ equals 2*v*_thres_ (Eq (11)), under optimal FvR profile conditions, $v_{0}^{opt}$ is a constant and does not depend on the absolute value of *snatch*_th_. Therefore, in theory, improvements in weightlifting performance solely may depend on increased theoretical maximal vertical barbell force capabilities (i.e., ${\bar{F}}_{0}^{opt}$) (Fig 4). This relation results in an improved force at *v*_thres_ and thus a higher barbell load that can be lifted. According to the example in Fig 4, at a constant *v*_thres_ of 2.0 m∙s^-1^, an increase in ${\bar{F}}_{0}^{opt}$ by +500 N is associated with an increase in *P*_max_ of +500 W that corresponds to an increase in ${snatch}_{th}^{max}$ of about +20 kg.

### Relative contribution of *P*_max_, *v*_thres_, and *s*_FvR_ on *snatch*_th_

**Fig 5:**
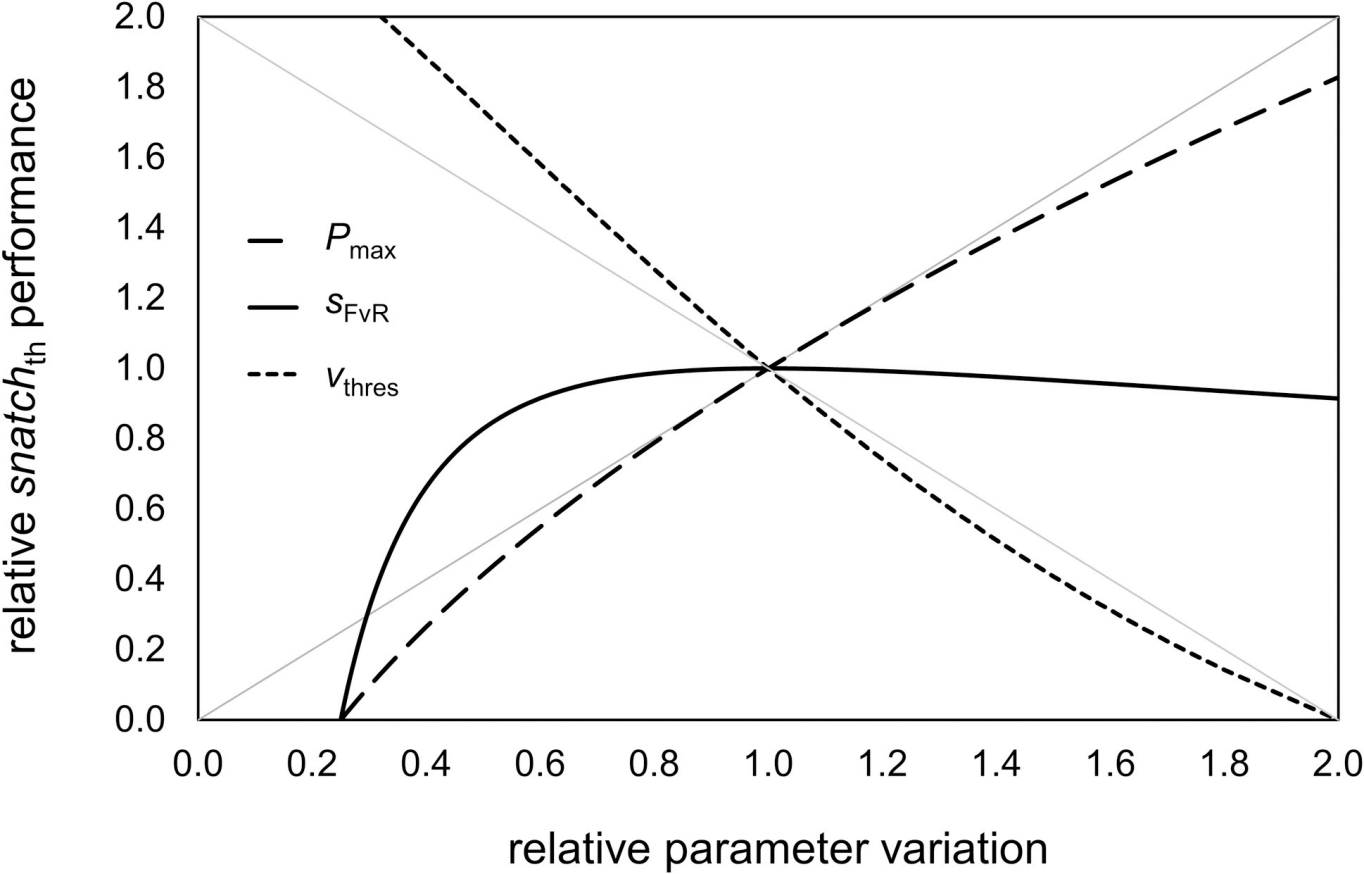
Relative changes in modelled 1RM snatch performance (*snatch*_th_) in response to relative parameter variation of vertical maximal barbell power (*P*_max_), slope of the barbell force–velocity relationship (*s*_FvR_), and vertical barbell threshold velocity (*v*_thres_). The reference parameter values (i.e., 1.0) are: *P*_max_ = 3000 W, *s*_FvR_ = –0.0011 m∙s^–1^∙N^–1^, *v*_thres_ = 1.85 m∙s^–1^. The distance of vertical barbell acceleration (*h*_acc_) is 0.8 m. Grey lines represent positive and negative identity lines.

The sensitivity analysis showed that relative *snatch*_th_ performance is primarily influenced by *P*_max_ and *v*_thres_ rather than *s*_FvR_ (Fig 5). Of note, Fig 5 shows that *P*_max_ and *v*_thres_ have a continuous positive or negative effect on *snatch*_th_, while *s*_FvR_ has its maximal effect at $s_{FvR}^{opt}$.

## Discussion

This study aimed to introduce the theoretical base of an optimal barbell snatch pull FvR profile (i.e., $s_{FvR}^{opt}$) to maximize theoretical snatch 1RM performance (${snatch}_{th}^{max}$) in weightlifting. We additionally examined the influence of $s_{FvR}^{opt}$, *P*_max_, and *v*_thres_ on *snatch*_th_ performance. In line with our study hypotheses, we observed at constant levels of *P*_max_ or *v*_thres_ that *snatch*_th_ is influenced by *s*_FvR_ and maximized at an optimal *s*_FvR_ value (i.e., $s_{FvR}^{opt}$). We further confirmed that changes in *P*_max_ and *v*_thres_ have a larger influence on *snatch*_th_ than changes in *s*_FvR_.

Competitive weightlifting requires both well developed technical skills and high mechanical power output to lift a maximal load in the overhead position in the snatch and the clean and jerk [25, 26]. The snatch pull FvR is an approach to quantify the external mechanical output at the barbell and to assess training related changes in *v*_0_, ${\bar{F}}_{0}$, *P*_max_ and *v*_thres_. While the parameters *v*_0_, ${\bar{F}}_{0}$, *P*_max_ are related to neuromuscular capabilities, *v*_thres_ is related to sport-specific technical skills. Our study findings revealed that besides training-induced improvements in neuromuscular capabilities, well-developed technical skills (i.e., amount of *v*_thres_) are needed and affect snatch performance. From the perspective of the neuromuscular capabilities, *P*_max_ presented the largest contribution to increase weightlifting performance. The leading effect of *P*_max_ to enhance ballistic performance is in agreement with evidence from the literature [27]. In addition, with an increased performance level (i.e., high *P*_max_), an optimal barbell FvR profile becomes more relevant to further maximize weightlifting performance. In this context, our simulations showed that the optimal barbell FvR profile is primarily force driven as *P*_max_ increases. This finding seems reasonable, given that the snatch 1RM is highly associated with the weightlifter´s maximal strength capabilities (i.e., 1RM squat) [25, 26, 28].

From the perspective of weightlifting technical skills, the simulations showed that changes in *v*_thres_ have a large influence on *snatch*_th_. For instance, if two lifters have the same *P*_max_ level to accelerate a maximal barbell load, the lifter with lower *v*_thres_ will achieve the higher snatch performance. In this context, Richter [29] postulated that increased maximal muscle strength and power have a larger impact to improve weightlifting performance than increased technical skills (ratio 10:1). In theory, however, if improved technical skills are associated with lowered *v*_thres_, the aforementioned ratio is ≈ 1:1.5 (Fig 5). Nevertheless, the large potential influence of *v*_thres_ on snatch performance should be put in perspective, since technical skills in weightlifting level off after 4–5 years of systematic training [30]. Therefore, elite weightlifters with a long history of systematic training (>5 years) are more likely to benefit from increased muscle strength and power (i.e., ${\bar{F}}_{0}$, *P*_max_) to improve snatch performance than from lowering *v*_thres_.

A few limitations of this study should be acknowledged. First, we should mention that the present findings are based on a biomechanical model that was verified using simulations. Although the concept of an optimal FvR to maximize performance seems to work for the vertical jump [10, 11], the validity for practical application in weightlifting has not yet been shown. Second, although the measurement error of FvR parameters (i.e., ${\bar{F}}_{0}$, *v*_0_, *P*_max_) derived from the snatch pull test is rather small [22], the measurement error for $s_{FvR}^{opt}$ and ${snatch}_{th}^{max}$ has not been assessed yet. However, knowledge of measurement error for $s_{FvR}^{opt}$ and ${snatch}_{th}^{max}$ is essential to guide training programming for the individual athlete. Finally, the modelled changes in snatch performance are based solely on changes in mechanical parameters during the acceleration phase (lift-off until maximal vertical barbell velocity) without accounting for the effect of an increased barbell load on the execution of the subsequent movement phases (i.e., turnover, catch, stand up) that also limit the performance outcome.

## Conclusions

Weightlifting performance can be improved through an increase in mechanical power output (*P*_max_), improved technical skills (*v*_thres_) and optimized FvR profile ($s_{FvR}^{opt}$). During long-term athlete development as well as in elite sports, improvements of *P*_max_ should be the main focus of training to further develop weightlifting performance [13, 17, 31]. In addition, a well-developed snatch technique enables weightlifters to efficiently lift loads at low *v*_thres_. This point can be declared as a major goal during the early stages of the long-term athlete development process [30]. For weightlifters with high *P*_max_ levels, the contribution of $s_{FvR}^{opt}$ to maximize snatch performance becomes more relevant. This finding is of great importance for elite weightlifters as the contribution of increased *P*_max_ and lowered *v*_thres_ to improve performance is strongly limited in world class athletes. Therefore, designing elite weightlifters training to achieve an optimal FvR profile has the potential to maximize performance. Even if optimization of the FvR profile can provide only small improvements in weightlifting performance, it may be of importance in competitions. As pointed out by Sandau and Lenz [32], on average, only 1.0% (≈ 3.7 kg) of total weightlifting performance (sum of snatch and clean and jerk) was the difference of making it to the podium or not (3^rd^ vs. 4^th^ place) at the Olympic Games.
